# Sustainable Valorization of Avocado By-Products: Green Extraction of Phenolics with NaDES and Their Use in Fresh-Cut Fruit Preservation

**DOI:** 10.3390/foods15101780

**Published:** 2026-05-18

**Authors:** Giulio Giannini, Jose Duvan Castillo Duque, Junior Bernardo Molina-Hernandez, William Royeiro Villamuez Benavides, Margarita María Andrade-Mahecha, Juan Felipe Grisales Mejia, Hugo Alexander Martinez-Correa, Silvia Tappi, Marco Dalla Rosa, Pietro Rocculi

**Affiliations:** 1 Department of Agricultural and Food Sciences, University of Bologna, 47521 Cesena, Italy; giulio.giannini3@unibo.it (G.G.); silvia.tappi2@unibo.it (S.T.); marco.dallarosa@unibo.it (M.D.R.); pietro.rocculi3@unibo.it (P.R.); 2 Departamento de Ingeniería, Facultad de Ingeniería y Administración, Universidad Nacional de Colombia Sede Palmira, Cr.32 # 12-00, Palmira 763536, Colombia; jocastillod@unal.edu.co (J.D.C.D.); wvillamuez@unal.edu.co (W.R.V.B.); mmandradem@unal.edu.co (M.M.A.-M.); jfgrisalesm@unal.edu.co (J.F.G.M.); hamartinezco@unal.edu.co (H.A.M.-C.); 3 Interdepartmental Centre for Agri-Food Industrial Research, University of Bologna, Via Q. Bucci 336, 47522 Cesena, Italy

**Keywords:** natural deep eutectic solvents (NaDES), avocado peel, phenolic compounds, green extraction, fresh-cut avocado, anti-browning

## Abstract

The fresh-cut avocado processing generates significant amounts of by-products, mainly peel and seed, with the peel representing a valuable source of phenolic compounds. In this context, the growing demand for sustainable technologies encourages the use of green solvents for bioactive compound recovery. In this study, natural deep eutectic solvents (NaDES) were evaluated as environmentally friendly solvents for the extraction of phenolic compounds from Hass avocado peels through ultrasound-assisted extraction and for their potential application in fresh-cut avocado. Phenolics were extracted using acidic water, ethanol, and NaDES based on choline chloride as a fixed hydrogen bond acceptor (HBA) and hydrogen bond donors (HBDs; lactic acid, glycerol, and citric acid) with the ultrasound-assisted system. The stability of the extracts was monitored for eight weeks (four weeks in darkness followed by four weeks under light exposure). Among the tested formulations, the lactic-acid-based NaDES showed the highest extraction efficiency and the best stability of phenolic compounds during storage (≥20 mg GAE g^−1^ dw during the storage period). The lactic-acid-based extract was then applied to fresh-cut avocado to evaluate its potential for antioxidant enrichment and browning prevention during refrigerated storage. The treatment increased phenolic content and contributed to improved color stability (during seven days of storage). Overall, lactic-acid-based NaDES represent a promising green solvent system for recovering phenolics from avocado peel and for their functional application in fresh-cut avocado within a circular valorization approach.

## 1. Introduction

Food waste is a pressing global issue with far-reaching environmental, economic, and ethical consequences. According to [1], in 2023 households were responsible for 53% of the total estimated food waste in Europe. The remaining 47% originated in earlier stages of the food supply chain, and within this share, primary production accounted for approximately 10% and food and beverage processing for about 19%, totaling 29% of food losses generated by the food industry [1]. Furthermore, it is estimated that the global agro-industrial chain generates 1.3 billion tons of food loss every year, and the amount of loss varies depending on the nature of the product [2].

In particular, the fresh-cut fruit industry is especially relevant to this topic, as fresh-cut or ready-to-eat fruit undergoes minimal processing, including washing, peeling, de-pitting, cutting, dipping, and packaging [3]. Therefore, depending on the product, the production process itself can result in up to a 30% loss of fresh weight [4].

However, this material is generally highly valuable, and both industry and researchers aim to shift from food loss to by-product valorization, extracting value from discarded biomass. In fact, plant material can contain nutrients with significant market potential because of their availability and low cost [5], including enzymes, vitamins, and antioxidants that can be recovered and used in a wide range of applications, such as food supplements, cosmetics, and pharmaceuticals [6].

Fresh-cut avocado is becoming a popular product to be sold at the wholesale level for restaurants, hotels, and catering services rather than retail. With over three million tons of avocados produced globally each year, and only the pulp typically consumed, the seeds and peel often become waste, highlighting the need for sustainable approaches to avocado by-products [7]; however, they are rich in phenolic acids, flavonoids, and tannins content, which are associated with antioxidant, antimicrobial, and anti-inflammatory properties [8]—the peel has been shown to contain almost twice the total phenolic content [9].

For the recovery of these molecules, traditionally, high-energy, solvent-intensive extraction methods, such as solid–liquid extraction (SLE), Soxhlet extraction, liquid–liquid extraction (LLE), and maceration [10], are used. However, the extraction process poses sustainability issues related to the type of solvents.

In this context, the principles of green chemistry and sustainable engineering have become increasingly important in developing new post-harvest technologies. One promising innovation in this field is the use of environmentally friendly solvents. Natural deep eutectic solvents (NaDES) have emerged as a green, low-cost alternative capable of providing high extraction yields, according to NaDES formulation, extraction technology, and product, in comparison with traditional solvents [11,12]. NaDES consist of a hydrogen bond donor and a hydrogen bond acceptor, forming a eutectic mixture with a melting point lower than that of the individual components, which make deep eutectic solvents some of the promising solvents and chemicals for sustainable material production [13,14].

They can be formulated using food-grade constituents, such as choline chloride, glycerol, glucose, or lactic acid, making them safe, non-volatile, and biodegradable [13]. Therefore, this extraction technique makes it possible to incorporate bioactive substances directly into food matrices for enrichment, without the need to separate the solvent from the extraction substrate [15].

Considering sustainability principles, several emerging technologies have been developed to enhance extraction efficiency, including ultrasound-assisted extraction (UAE), microwave-assisted extraction (MAE), and pressurized liquid extraction (PLE) [16]. Among these, UAE is popular, as it is unexpensive, efficient, and non-thermal [17]. The combination of NaDES and UAE has been explored before on other products, including blueberry leaves [18], propolis [19], and artichoke outer petals [20], among others.

Given this context, the valorization of avocado by-products through green extraction technologies represents a promising strategy to recover high-value bioactive phenolic compounds while reducing food loss. Nevertheless, the optimal combination of solvent composition and extraction conditions remains largely product-specific, and only few studies have focused on avocado peel and pit [9,21]. Recent studies have shown that NaDES can improve not only the extraction efficiency, but also the storage stability of phenolic-rich extracts from different plant by-products, including orange by-products, bilberry, blueberry peel, and *Helichrysum arenarium* [22,23,24,25]. This stabilizing effect has been mainly related to the hydrogen bond network of NaDES, which may reduce molecular mobility and limit oxidative degradation, although it strongly depends on solvent composition, phenolic class, and storage conditions.

In addition, NaDES extracts can potentially be used directly in food systems without solvent removal, allowing their application as ready-to-use functional ingredients [26,27]. This approach has been explored for food fortification, such as cocoa by-product extracts in chocolate milk and hazelnut cuticle extracts in plant-based beverages, as well as for food preservation through NaDES-based coatings for strawberries [27,28,29]. However, most studies still address extraction efficiency, extract stability, and food application separately. Limited information is available on approaches combining NaDES-based extraction from avocado by-products, storage stability of the resulting phenolic extracts, and their direct application in a fresh-cut fruit system.

This gap is particularly relevant for fresh-cut avocado, where phenolic enrichment may be technologically challenging because phenolic compounds can act as antioxidants but also as substrates for polyphenol-oxidase-mediated browning. Therefore, this study aims to build upon previous works [9,21] by advancing the valorization of avocado peel through ultrasound-assisted NaDES extraction, evaluating the stability of the obtained phenolic extracts during storage under dark and light exposure, and assessing the potential of the most effective extract for phenolic enrichment and color preservation in fresh-cut avocado.

## 2. Materials and Methods

### 2.1. Avocado Peel Flour

Hass avocado fruits (*Persea americana Mill*. var. Hass) were purchased from a supermarket in Palmira, Valle del Cauca, Colombia. The fruits were selected based on their ripeness stage (green epicarp) and the minimum quality requirements specified in NTC 1248, which state that avocados must be whole, intact, clean, free of foreign odors or flavors, and free of damage caused by low temperatures or mechanical impact. The fruits were stored at 28 °C until they reached consumption ripeness (black epicarp). The fruit was manually prepared by separating the epicarp, seed, and pulp. Each fraction was then placed in an ultra-low-temperature freezer (Ecofríal ICC—210E, China) at −40 °C until use. The peel was then dried in a forced-convection oven (Memmert UF 55, Schwabach, Germany) with a constant airflow at 50% speed and temperature of 80 °C.

Drying was considered completed when the sample reached a moisture content below 7% (0.75 h). The dried samples were then ground in a mill (Hamilton Beach, Southern Pines, NC, USA) until a fine powder was obtained. Drying temperature was selected in preliminary trials aimed at evaluating the higher phenolic extraction. The flour was sent to the laboratory of Bologna University where it was characterized for particle size, color, pH, and water activity and used for the following extraction process.

### 2.2. Peel Flour Extract

#### 2.2.1. NaDES Preparation

NaDES were prepared according to the methodology reported by [9]. Choline chloride was used as the fixed hydrogen bond acceptor (HBA), while lactic acid (Lac), glycerol (Gly), and citric acid (Cit) were evaluated as hydrogen bond donors (HBDs). Each pair of components was mixed and heated to 70 °C under continuous stirring until a homogeneous liquid was obtained. The molar ratios were as follows: choline chloride:lactic acid (1:2), choline chloride:glycerol (1:2), and choline chloride:citric acid (1:1). Water was added to adjust the final composition so that choline chloride represented 27% of the total solution. As controls, distilled water (Wa) and ethanol (80%; Et) were used. Both solutions were added with 0.5% acetic acid to maintain an acidic pH and stabilize phenolic compounds.

#### 2.2.2. Ultrasound-Assisted Extraction (UAE)

Extractions were performed in a 25 mL glass extraction vessel equipped with an external jacket connected to a thermostatic circulating bath (Jeio Tech lab, Geumcheon-gu, Seoul, Republic of Korea ) for temperature control. The extraction temperature was maintained at 40 °C to promote extraction while preventing thermal degradation of phenolic compounds.

Ultrasound-assisted extraction was carried out using an ultrasound processor (Hielscher Ultrasound Technology, Teltow, Germany) set to 40% amplitude and 45 kHz frequency, with a 0.2 cm-diameter probe. A total of 1.0 g of sample was placed into the extraction vessel, followed by the addition of 25 mL of NaDES. Extraction was performed for 10 min. The sample-to-solvent ratio and extraction time were previously optimized through preliminary tests. The extract was filtered through a stainless-steel fine-mesh sieve (110 mm) to remove the powder and collected in falcon tubes.

### 2.3. Light Influence on Phenolic Compounds During Extract Storage

In order to evaluate the effect of the light on the stability of phenolic content, the extracts were stored for 4 weeks at room temperature (25 °C) in the dark, and then for an additional 4 weeks at room temperature (25 °C) under light exposure, 6500 °K ( T8G36R18-65- DND, 18 W/965, Philips, Cambridge, MA, USA). Extracts were analyzed for total phenolic content (TPC). All measurements were performed in triplicate. On selected samples, the phenolic profile was analyzed.

### 2.4. Avocado Fresh-Cut Application: Antioxidant Enrichment and Color Preservation

The best performance combination of the extraction (choline chloride + lactic acid; LacEx) was used to evaluate the antioxidant enrichment and anti-browning effect of fresh-cut avocado. The extraction was carried out as in Section 2.2.2, with the only modification being using 0.5 g in 25 mL of solvent according to a range reported in previous research [30].

Fruits were sanitized in 200 ppm sodium hypochlorite for 2 min, dried, and cut into four quarters. Each fruit was used as an experimental block and divided into four quarters, which were randomly assigned to one of the four treatments. For each sampling day, four independent fruits were used, resulting in four biological replicates per treatment and day. The treatments consisted of immersion for 2 min in distilled water (Wa; acetic acid 0.5%), distilled water (acetic acid 0.5%) with phenolic extract (WaEx), lactic-acid-based NaDES (Lac), and lactic-acid-based NaDES with phenolic extract (LacEx). After treatment, quarters were air-dried for 15 min and later were conditioned in an aluminum container (590 mL and 187 × 137 mm, respectively) wrapped with a 20-micron-thick film packaged in fresh-cut trays package headspace (2 cm).

Samples were stored at 4–6 °C and analyzed at 0, 1, 4, and 7 days of storage. Two experimental sets were defined. The first set consisted of 4 fruits and was used for non-destructive analyses (color and image analysis), with the same samples monitored throughout storage. The second set consisted of 12 fruits and was used for destructive determination of total phenolic content (TPC), with 4 fruits analyzed at each sampling day (0, 1, and 4). At day 7, samples from the first set were also used for TPC analysis.

### 2.5. Analytical Determinations

#### 2.5.1. Particle Size

The flour particle size distribution was measured with Mastersizer 3000 (Malvern Instruments Ltd., Malvern, UK) according to [31]. A representative portion of sample (5 g) was injected with air as a carrier gas. Particle size was evaluated by the parameter D90 (μm), which represents particle diameter corresponding to 90% of the cumulative distribution.

#### 2.5.2. Color, Water Activity, and pH

Water activity and pH were measured according to the AOAC methods 981.12 and 925.1 [16]. Water activity (aw) was determined using a hygrometer AquaLab CX 4-TE (Decagon Devices Inc., Pullman, WA, USA). The pH values were determined with a laboratory pH meter (MP220, Mettler Toledo International, Polaris Parkway, OH, USA) directly in the extracts. All measurements were performed in triplicate.

The color analysis was performed using a Konica Minolta Chroma Metre CR-5 spectrophotocolorimeter (Konica Minolta, Osaka, Japan) equipped with a D65 light source. Measurements were performed directly on a target mask with a measuring area of 8 mm using standard 10° observers. For each sample, 10 different halves were analyzed, and for each half the CIELab colorimetric coordinates were recorded in 2 different areas. The coordinates L* (luminosity), a* (greenness), and b* (yellowness) were analyzed. All measurements were performed at least in triplicate.

#### 2.5.3. Total Phenol Content (TPC)

Total phenol content (TPC) was assessed following the Folin–Ciocalteu method [32] with some modifications. Briefly, 100 µL of extract was transferred into a Falcon tube, followed by the addition of 2.8 mL of sodium carbonate solution (8%) and 100 µL of Folin–Ciocalteu reagent (2 N). The mixture was brought to a final volume of 10 mL with distilled water and incubated in darkness at room temperature for 30 min. Absorbance was measured at 720 nm using a Shimadzu spectrophotometer model UV-1601 (Shimadzu, Kyoto, Japan), and gallic acid was used to construct the calibration curve. Results are expressed as milligrams of gallic acid equivalents per gram of fresh weight (mg GAE/g). TPC was assessed every 7 days for 8 weeks. All measurements were performed in triplicate.

#### 2.5.4. Phenolic Profile (HPLC)

The quantification was carried out according to the methodology reported by [33]. HPLC analysis was performed on a Shimadzu (LC-2030 LT Series-i, Long Beach, CA, USA) equipped with a photodiode detector, solvent degasser, quaternary pump, autosampler with temperature control, and thermostat column compartment. The separation was achieved using a C18-phenyl column (4.6 × 50 mm, 2.5 µm particle size; Xbridge^®^, Waters, Milford, MA, USA) protected with a security guard obtained from Phenomenex (AJ0-8788, Phenomenex, Torrance, CA, USA). The procedure consisted of acidified water (water/formic acid, 99.9:0.01 *v*/*v*; solvent A) and acidified acetonitrile (acetonitrile/formic acid, 99.9:0.01 *v*/*v*; solvent B). The optimized linear gradient was as follows: 0–8 min, 2% B; 8–37 min, 10% B; 37–40 min, 0% B; 10 min, 2% B. The flow rate was 0.8 mL/min, and the temperature was 60 °C. The detector acquisition was 190–800 nm: 280 nm for catechin, epicatechin, vanillic acid, and p-hydroxybenzoic acid, 327 nm for caffeic acid, and 365 nm for quercetin, luteolin, naringin, and rutin. The calibration curves for caffeine, theobromine, catechins, and dimer B2 were made from commercially available analytical standards (R^2^ = 0.99). All the results are expressed as ug of sample per g of avocado peels (dry matter basis).

#### 2.5.5. Image Analysis

Digital color images of avocado slices were acquired following the methodology of Schouten et al. (2020) using a D7000 digital camera (Nikon, Tokyo, Japan) equipped with a 105 mm lens (AF-S Micro Nikkor, Nikon, Japan) [34]. The camera was placed inside a dark chamber to eliminate ambient light interference, under controlled illumination provided by four daylight fluorescent lamps (TL-D D.N.D., 18 W/965, Philips, USA) with a color temperature of 6500 K (D65 standard). The 60 cm fluorescent tubes were positioned 35 cm from the sample at a 45° angle. Camera settings were manually configured as follows: shutter speed 1 s, aperture f/18, and exposure 0 EV.

Image analysis was performed using FIJI/ImageJ® software (Version 1.51n, NIH, Bethesda, MD, USA). to extract relative L*, a*, and b* values and quantify browning development. Delta E (ΔE) and browning area (BA) were calculated, respectively, as shown in Equations (1) and (2):

(1) $$\Delta E=\sqrt{{\left({L_{n-}L}_{0}\right)}^{2}+{\left({a_{n-}a}_{0}\right)}^{2}+{\left({b_{n-}b}_{0}\right)}^{2}}$$

(2) $$BA\;\left(\%\right)=\frac{Browning\;surface}{Total\;surface}\times 100$$

### 2.6. Statistical Analysis

All analyses were performed in triplicate and expressed as mean ± SD. All data were subjected to one-way ANOVA for mean comparisons and standard deviation (SD), and significant difference means at a significance level of *p* ≤ 0.05 were calculated according to the Tukey HSD post hoc test. Data were processed using XLSTAT (XLSTAT 2020.1.3.65324; Addinsoft, New York, NY, USA) software.

## 3. Results and Discussion

### 3.1. Flour Characterization

The physicochemical characterization parameters are shown in Table 1.

The color was characterized by a low L* value (40.81 ± 0.02), indicating a dark appearance, together with relatively high a* (11.38 ± 0.03) and b* (27.24 ± 0.12) values, indicating a predominance of red and yellow components, respectively. Overall, these color coordinates describe a dark brown appearance.

The particle size parameters indicate a certain degree of variability in particle size, as reflected by the substantial distance between D10 and D90 and by the D90/D10 ratio (≈3.3) [35], which could affect variability during the extraction process. However, 90% of the particles are smaller than 736.6 ± 24.9, indicating a relatively fine powder suitable for efficient extraction.

### 3.2. Phenolic Extracts: Stability and Profile

**Figure 1:**
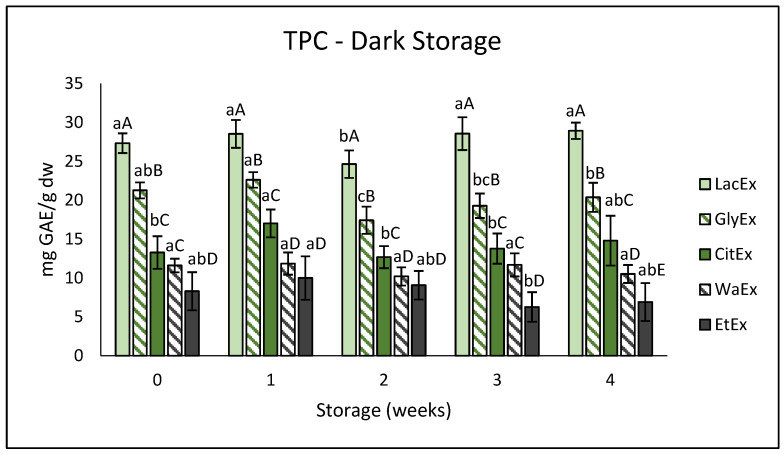
Total phenolic content (TPC) of the different extracts during dark exposure storage. Values are expressed as mg GAE g^−1^ dw and reported as mean ± standard deviation. Different lowercase letters indicate a significant difference between the same extract during storage weeks (*p* < 0.05), while different uppercase letters indicate significant differences between different treatments within the same storage week (*p* < 0.05).

In order to develop a sustainable extraction method to recover phenolic compounds from avocado peel using environmentally friendly solvents, different combinations were tested using choline chloride as the fixed hydrogen bond acceptor (HBA), while lactic acid (LacEx), glycerol (GlyEx), and citric acid (CitEx) were used as hydrogen bond donors (HBDs). These systems were also compared with traditional solvents, namely, distilled water (H_2_O) and 80% ethanol (EtOH). A significant difference among the treatments was observed (*p* ≤ 0.05; Figure 1).

Considering the initial values of TPC, the use of different solvents proved to have a significant effect on the extraction yield. Among the NaDES, the use of Lac allowed us to obtain a concentration of 28 ± 0.2 mg GAE/g, significantly higher compared to Gly (20 ± 0.3) and Cit (14 ± 0.3). Conventional solvents showed lower performances. Extraction with water led to values similar to the ones obtained with citric acid, while ethanol showed a lower extraction capacity, with a TPC of 8 ± 0.4. These results are consistent with previous studies, which have consistently reported NaDES as more effective solvents than conventional ones for the extraction of phenolic compounds under comparable conditions and across a wide range of food matrices, not limited to avocado waste [36,37,38]. In detail, our study achieved phenolic concentrations approximately six-fold higher than those obtained from pomegranate peel (4.14 mg EAG mL^−1^), and values comparable to those observed for sour cherry pomace and olive oil extracts analyzed in this work. These findings further highlight avocado by-products as a promising and rich source of phenolic compounds [36,37,38].

The different performances of the NaDES formulation are mainly attributed to differences in pH, viscosity, density, and polarity [9]. In particular, density and viscosity may influence the distribution of the extracted powder within the solvent. In principle, a denser solvent could be expected to provide higher extraction efficiency and, therefore, CitEx and GlyEx would be expected to yield higher phenolic content compared to LacEx [39]. However, in the present system, it was noticed that higher viscosity and density lead to the flotation of the powder on top of the solvent, resulting in a less homogeneous mixing during the ultrasound extraction. In contrast, in the LacEx the powder was able to deposit at the bottom of the extracting glass chamber, which likely promoted a more effective mixing during extraction. However, this does not fully explain the difference observed between CitEx and GlyEx. According to Fuad et al. (2021) [39], the polarity of GlyEx is approximately twice that of both LacEx and CitEx, which may favor phenolic solubilization and partially explain the observed extraction trend. Finally, the lower suitability of water and ethanol for phenolic extraction compared with NaDES has been widely reported in the literature [11].

#### 3.2.1. Total Phenol Content: Dark Storage

Reference [22] observed, in more detail, TPC preservation in NaDES extracts from orange by-products compared with ethanolic extracts during storage. Our results revealed that NaDES are efficient for the extraction of bioactive compounds from by-products, and these extracts can represent an alternative for the food industry to enrich food products with natural ingredients.

#### 3.2.2. Total Phenol Content: Light Exposure

**Figure 2:**
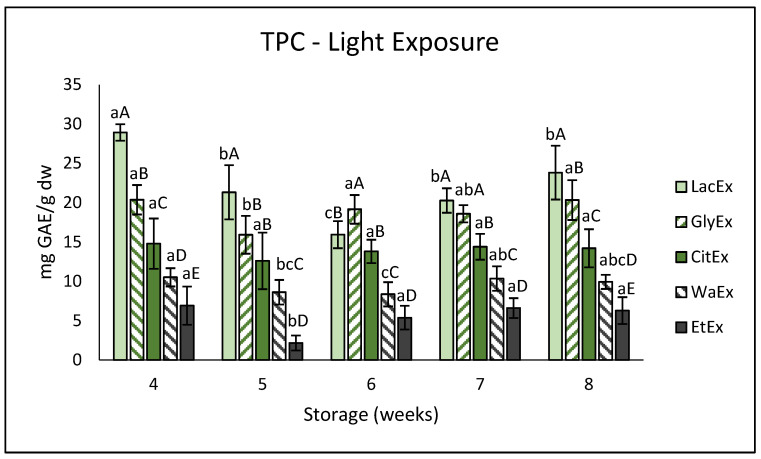
Total phenolic content (TPC) of the different extracts during light exposure storage. Values are expressed as mg GAE g^−1^ dw and reported as mean ± standard deviation. Different lowercase letters indicate a significant difference between the same extract during storage weeks (*p* < 0.05), while different uppercase letters indicate significant differences between different treatments within the same storage week (*p* < 0.05).

The same samples were then stored under light exposure (weeks 5–8) to accelerate shelf-life deterioration, and the results are shown in Figure 2.

Compared with dark storage, light exposure led to higher variability in TPC values, suggesting that phenolic compounds were exposed to more stressful storage conditions. Previous studies have shown that light can affect phenolic stability depending on the phenolic class, temperature, and solvent/matrix composition. In NaDES-based bilberry anthocyanin extracts, Jovanovic et al. [25] reported only limited differences between storage at 25 °C in darkness and under natural light, suggesting a protective effect of the NaDES system against light-induced degradation [25]. Conversely, studies on grape stem and grape cane extracts showed that light can accelerate the degradation of specific phenolic compounds, especially when combined with higher temperature [40,41].

In the present study, LacEx and GlyEx showed the highest TPC values under light exposure, although their relative performance was less consistent than under dark storage. GlyEx was significantly higher than LacEx at week 6 (*p* ≤ 0.05), while no significant difference was observed at week 7. However, LacEx showed the highest TPC both at the beginning of light exposure (week 5) and at the end of storage (week 8), suggesting a more robust overall performance.

The fluctuations observed during light exposure may reflect the combined effects of irradiation, a possible temperature increase, and solvent-dependent interactions with phenolic compounds [42]. Moreover, since the Folin–Ciocalteu assay measures overall reducing capacity rather than individual phenolic molecules, changes in TPC may also include the contribution of phenolic transformation products. Overall, although LacEx showed a moderate decrease over time, it remained the extract with the highest phenolic content after eight weeks, supporting the suitability of lactic-acid-based NaDES for preserving phenolic reducing capacity during storage.

#### 3.2.3. Phenolic Profile Changes

**Figure 3:**
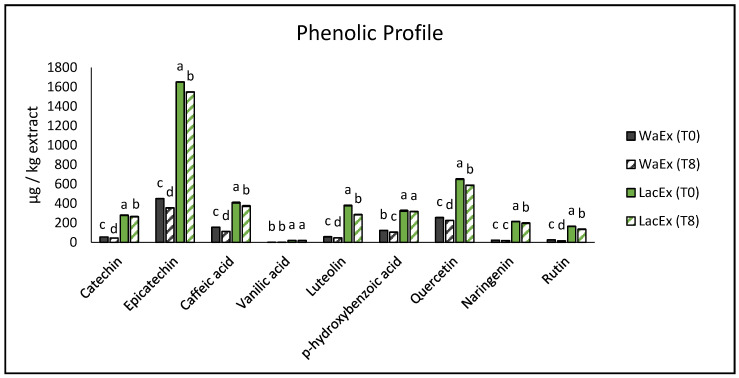
Phenolic profile of LacEx and WaEx at week 0 (T0) and week 8 (T8). Individual phenolic compounds (catechin, epicatechin, caffeic acid, vanillic acid, luteolin, p-hydroxybenzoic acid, quercetin, naringenin, and rutin) are expressed as µg kg^−1^ of extract and reported as mean ± standard deviation. Different lowercase letters indicate significant differences within the same phenolic compound (*p* < 0.05).

In order to identify phenolic compounds in avocado peel extract, the extract obtained in the most performant condition (LacEx) was analyzed through the HPLC method previously described. LacEx, as the best solvent, was characterized more deeply through HPLC at week 0 and week 8 of storage and compared with WaEx as the control. The results are shown in Figure 3.

The HPLC results confirm the trend observed in the TPC analysis. All the analyzed phenolic compounds were significantly higher in the lactic-acid-based NaDES extract (LacEx) than in the water extract (WaEx). Moreover, most compounds showed the same pattern, with significantly higher values at week 0 compared to week 8.

However, the decrease was moderate, indicating good stability of the phenolic extract during storage for both WaEx and LacEx. The most abundant recovered phenolic acid was epicatechin, which decreased from 1652 ± 1.56 to 1548 ± 1.89 µg/kg at week 8 for LacEx and from 450 ± 0.15 to 356 ± 1.5 µg/kg for WaEx. On the other hand, catechin was much less recovered and decreased from 280 ± 2.01 to 265 ± 3.21 µg/kg for LacEx and from 56 ± 0.23 to 45 ± 0.16 µg/kg for WaEx. These results are in accordance with [43], who were not able to quantify catechin but were able to quantify a large amount of epicatechin in avocado by-products.

The other quantified phenolic compounds also showed moderate decreases during storage, although their concentrations remained consistently higher in LacEx than in WaEx. Among these compounds, quercetin showed a slight decline over time, decreasing from 652 ± 2.59 to 589 ± 1.58 µg/kg in LacEx (9.6% reduction) and from 256 ± 1.25 to 226 ± 1.15 µg/kg in WaEx (11.7% reduction). Quercetin and its derivatives are commonly reported among the major flavonols in avocado peel, as also described by Kosińska et al. (2012) [44], who reported significant levels of quercetin derivatives in dried avocado peel extracts.

A similar trend was observed for caffeic acid, which decreased from 410 ± 5.21 to 375 ± 4.32 µg/kg in LacEx (8.5% reduction) and from 156 ± 0.65 to 112 ± 0.65 µg/kg in WaEx (28.12% reduction) during storage. Caffeic acid has also been previously detected in avocado peel extracts, although often at lower concentrations. For example, Ref. [43] reported caffeic acid values of approximately 4.1 ± 1.1 µg/kg, highlighting the variability that can occur depending on extraction conditions, raw material, and analytical methods.

Flavonoids such as luteolin and naringenin also followed the same pattern, showing moderate decreases between week 0 and week 8. Luteolin decreased from 380 ± 3.26 to 286 ± 1.78 µg/kg in LacEx (24.7% reduction) and from 58 ± 0.82 to 46 ± 0.15 µg/kg in WaEx (20.6% reduction), while naringenin decreased from 215 ± 2.18 to 198 ± 4.23 µg/kg in LacEx (7.9% reduction) and from 23 ± 0.25 to 18 ± 1.32 µg/kg in WaEx (21.7% reduction). Although these compounds are typically reported at relatively low concentrations in avocado peel, they are important contributors to the antioxidant and bioactive properties of the extracts [43].

Finally, rutin, a glycosylated derivative of quercetin, also showed a slight decrease during storage, from 165 ± 1.56 to 135 ± 2.56 µg/kg in LacEx (18.1% reduction) and from 26 ± 0.15 to 15 ± 1.15 µg/kg in WaEx (42% reduction). High rutin levels in avocado peel extracts have been reported when deep eutectic solvents are used as extraction media, in contrast with our findings [45].

Exceptions to this trend were vanillic acid and p-hydroxybenzoic acid. In the case of vanillic acid, the very low recovery levels made it difficult to detect significant differences during storage, with values of 20 ± 1.26 µg/kg at week 0 and 19.5 ± 1.89 µg/kg at week 8 (2.5% reduction). Similarly, p-hydroxybenzoic acid did not show significant variations over time in LacEx, with concentrations of 326 ± 5.24 µg/kg at week 0 and 318 ± 1.78 µg/kg at week 8 (2.4% reduction). The low abundance of these compounds in avocado peel extracts is consistent with previous studies. For example, Ref. [43] reported very low concentrations of these phenolic acids in avocado peel extracts, with values of approximately 0.2 µg/kg for vanillic acid and 3.8 µg/kg for p-hydroxybenzoic acid, confirming that these compounds typically occur at much lower levels than flavonoids and flavan-3-ols in this matrix.

These results suggest that lactic-acid-based NaDES are suitable for maintaining phenolic extract stability over time, in agreement with previous reports [39]. The ability of NaDES to preserve extracted compounds has been attributed to the strong hydrogen bond network formed within the solvent system, which increases viscosity and reduces solute mobility, thereby potentially enhancing stability.

### 3.3. Fresh-Cut Avocado Application

From the previous test (Section 3.2), LacEx was chosen as the best solvent for phenolic recovery. This work is proposing the application of LacEx as a way to enrich fresh-cut avocado. However, phenols are the substrate of the polyphenol oxidase (PPO), which catalyzes the browning reaction. Therefore, a color evaluation was carried out in order to understand if the natural anti-browning effect on lactic-acid-based NaDES (Lac) [13] was sufficient to neutralize the oxidation of the supplemented phenols, along with total phenol assessment.

#### 3.3.1. Image Analysis: Color Evaluation

**Figure 4:**
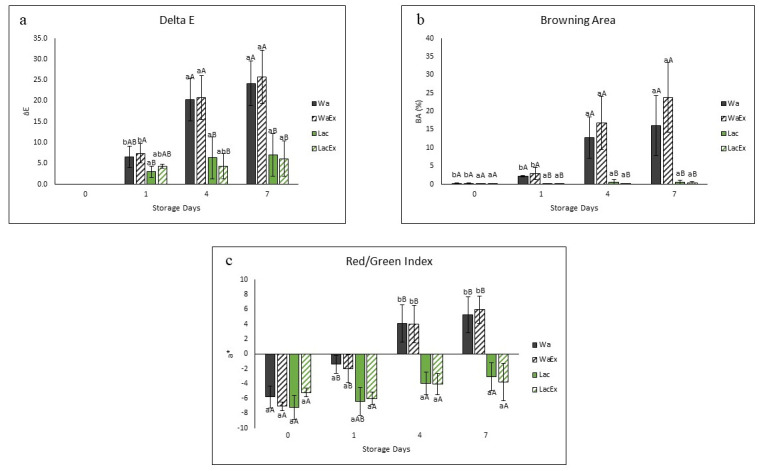
Color difference (ΔE) (**a**), percentage of browning area (BA) (**b**), and changes in the a* (red/green) coordinate (**c**) of samples treated with water (Wa), water extract (WaEx), lactic-acid-based NaDES (Lac), and lactic-acid-based NaDES extract (LacEx) during storage (days 0, 1, 4, and 7). Values are expressed as mean ± standard deviation. Different lowercase letters indicate significant differences among storage days within the same treatment, whereas different uppercase letters indicate significant differences among treatments within the same storage day (*p* < 0.05).

**Figure 5:**
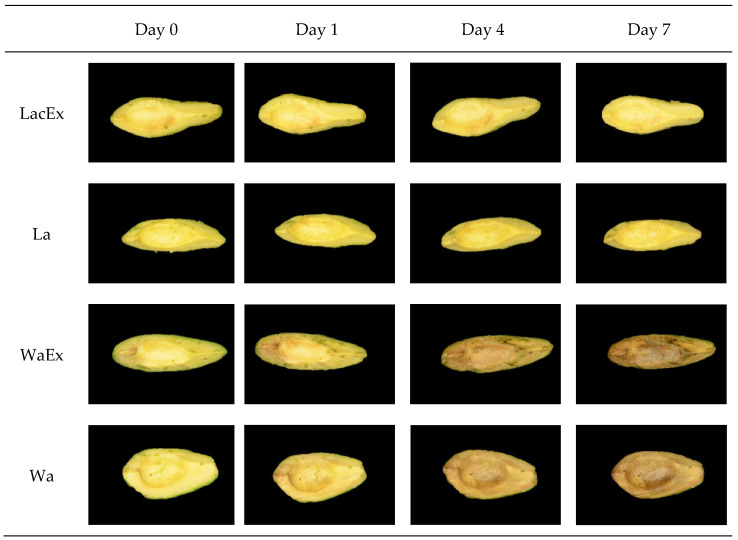
Changes in appearance of fresh-cut avocado during storage under different treatments.

The results of color difference (ΔE), percentage of browning area (BA), and changes in the a* (red/green) coordinate are shown in Figure 4. Visual changes in appearance of fresh-cut avocado during storage under different treatments are shown in Figure 5.

The avocado halves’ color slightly changed after one day of storage; however, the browning process occurred more markedly after day 4. The ΔE analysis showed minimal color differences after one day of storage between the treatments, with only Lac being significantly lower (*p* ≤ 0.05) than the other treatments. After four days, two distinct groups were observed, with Wa and WaEx showing significantly (*p* ≤ 0.05) higher ΔE values than Lac and LacEx, respectively. The same pattern was maintained at day 7, with an even greater separation between the two groups. During storage, Wa and WaEx both showed significantly (*p* ≤ 0.05) higher ΔE values at days 4 and 7 compared to day 1 (Figure 5). In contrast, Lac and LacEx did not show significant (*p* ≤ 0.05) differences throughout the storage period, suggesting the protective effect of lactic-acid-based NaDES. The same trend was confirmed by the percentage of browning area (BA). The two groups, Wa/WaEx and Lac/LacEx, were already significantly different (*p* ≤ 0.05) at day 1. The separation between the groups increased further at day 4 and became even more pronounced at day 7, again indicating improved color preservation in the Lac/LacEx treatments.

The Wa/WaEx group showed a significant increase (*p* ≤ 0.05) at days 4 and 7 compared to days 0 and 1. At these time points, fruit variability resulted in higher standard deviations, making it more difficult to detect further differences among storage days. In contrast, Lac/LacEx did not show significant differences (*p* ≤ 0.05) throughout storage, and the treatments appeared to overcome fruit variability, resulting in markedly lower standard deviations. The a* coordinate indicates a tendency toward green when values are negative and toward red when values are positive. Browning in avocado flesh involves a shift from the natural green color toward brown, which is associated with an increase in the red component. Therefore, the a* value serves as a useful visual indicator of browning.

The trend in a* values was consistent with the previous color analyses. The treatments were clearly divided into two clusters: Wa/WaEx and Lac/LacEx. Significant differences between the two groups were already observed at day 1 and became more pronounced at days 4 and 7. In particular, Lac/LacEx retained the green component, remaining consistently negative throughout storage. In contrast, Wa/WaEx progressively lost the green component from day 4 onward, shifting toward positive values and indicating an increase in the red component.

These color analysis results suggest that the lactic-acid-based NaDES may exert a protective antioxidant effect [39], contributing to color preservation over time. Whether Lac acts as a PPO inhibitor or is able to reduce quinones back to phenols [46] remains unclear and is not well documented in the literature. Măntăilă et al. reported that a NaDES extract from grape pomace exerted an uncompetitive inhibitory effect on PPO, which was mainly associated with its richness in small phenolic acids. However, the phenolic profile of the present extract was markedly different, being dominated by epicatechin, that may also act as a PPO substrate [47]. Therefore, the same inhibitory mechanism cannot be directly assumed for the present system. Since enzymatic browning in fresh-cut avocado is mainly mediated by polyphenol oxidase (PPO), evaluating the direct inhibitory effect of the extract on PPO activity would help clarify whether the observed color preservation is associated with enzyme inhibition, as previously reported for other avocado-derived anti-browning extracts.

Since PPO has an optimal activity at pH around 5–7 [48], the lower pH of the extract may have contributed to the reduced browning observed. However, Wa/WaEx were also adjusted with 0.5% acetic acid to promote phenolic stability; therefore, pH alone does not fully explain the differences among treatments. According to Ref. [49], the structure of PPO may even be stabilized in the presence of betaine-glycerol-based NaDES, possibly due to an increase in α-helix content in the enzyme secondary structure. Moreover, NaDES interactions with PPO were reported not to affect the active site or its substrate-binding capacity [49]. Therefore, based on the present results, a plausible explanation for the ability of Lac/LacEx to limit enzymatic browning may be the scavenging capacity of the NaDES system [39], potentially acting through a mechanism similar to that proposed for ascorbic acid, i.e., the donation of electrons and protons to quinones or o-diphenols during the PPO reaction, thereby preventing quinone polymerization into melanins [50].

#### 3.3.2. Total Phenol Content (TPC)

**Figure 6:**
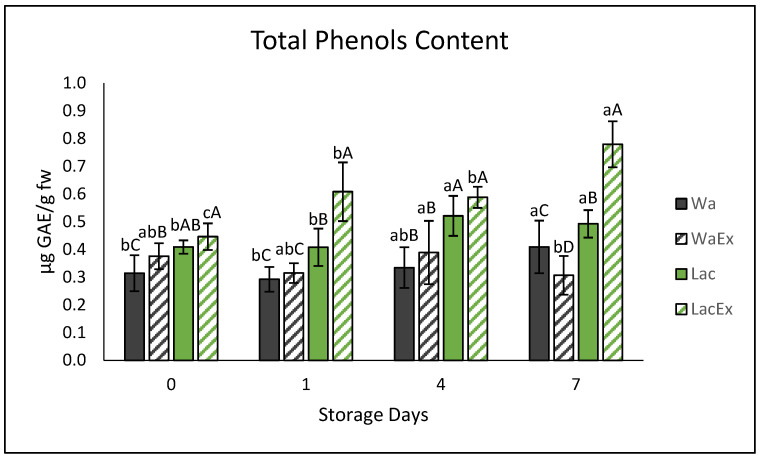
Total phenol content (TPC) of samples treated with water (Wa), water extract (WaEx), lactic-acid-based NaDES (Lac), and lactic-acid-based NaDES extract (LacEx) during storage (days 0, 1, 4, and 7). Values are expressed as mean ± standard deviation. Different lowercase letters indicate significant differences among storage days within the same treatment, whereas different uppercase letters indicate significant differences among treatments within the same storage day (*p* < 0.05).

The TPC of fresh-cut avocado is shown in Figure 6.

After treatment application (day 0), differences among treatments were not highly pronounced (*p* ≤ 0.05); however, LacEx was already significantly higher (*p* ≤ 0.05) than Wa and WaEx. However, it should be considered that TPC does not directly quantify phenolic compounds, but rather the overall reducing capacity of compounds able to oxidize the Folin–Ciocalteu reagent [51]. Although phenolic compounds are the main contributors, other antioxidant substances may also react, such as NaDES. On this matter, previous tests confirmed that the formulated NaDES did not interfere with the assay.

However, because ethanol was used to extract phenols, it is probable that leftover Lac after the process improved the ability to extract phenolics that were already in the avocado cells, which accounts for the elevated levels of Lac compared to, specifically, WaEx.

During storage, Wa and WaEx showed limited variation. Wa exhibited a slight but significant (*p* ≤ 0.05) increase at days 4 and 7, while WaEx remained relatively stable, with a small but significant decrease at day 7. In contrast, LacEx showed markedly higher TPC (*p* ≤ 0.05) values at days 4 and 7, with a progressive increase over time. A similar increasing trend was also observed for Lac.

One possible explanation is that the presence of NaDES may enhance the solubility and availability of phenolic compounds during extraction, as mentioned earlier. That said, fresh-cut fruits are known to synthesize phenolic compounds as a stress response following cutting, through activation of the phenylalanine ammonia-lyase (PAL) pathway. PAL catalyzes the conversion of L-phenylalanine into trans-cinnamic acid, the first precursor in the biosynthesis of phenolic acids, flavonoids, tannins, and lignin [52]. Therefore, an increase in phenolic content over time could be expected in fresh-cut samples [53]. However, in Wa and WaEx, newly synthesized phenolics may have been partially oxidized by PPO and converted into melanins, counteracting their accumulation [54]. In contrast, Lac and LacEx may have acted as antioxidant and color-stabilizing systems (as explained in Section 3.3.1), preserving phenolic compounds during storage.

Overall, the proposed explanation for phenolic accumulation in Lac is related to its ability to maintain the phenolic antioxidant system of fresh-cut avocado, while in LacEx it is related to the provided additional enrichment of the extract, resulting in more than a two-fold increase in TPC compared to Wa and WaEx on day 7. Although LacEx showed promising effects in terms of color preservation and phenolic enrichment, the potential sensory impact of NaDES-based treatments should be carefully considered. Recent reviews have highlighted that, despite the promising role of NaDES as green solvents, carriers, antioxidants, and color-stabilizing systems in food applications, their direct use still requires further evaluation regarding sensory acceptability, safety, regulatory aspects, and interactions with complex food matrices [55]. This aspect is particularly relevant since NaDES are often not removed from the final extract, meaning that even low residual concentrations may influence taste, aroma, texture, or mouthfeel. Despite significant progress in the chemical and microbiological characterization of NaDES-treated foods, their sensory acceptability remains insufficiently documented. In particular, lactic acid, at the concentration used in this study, may introduce a distinct tangy, sour, or mildly fermented flavor and aroma [55]. However, this sensory impact is generally manageable, and at appropriate levels it may even contribute to acceptable or improved flavor profiles compared to other compounds. Recent studies have demonstrated the effectiveness of choline-chloride-based NaDES in recovering phenolic compounds from agro-industrial by-products. For example, Ran et al. reported optimized flavonoid extraction from grape seeds using a choline:lactic acid (1:2) system, with 92% antioxidant activity retention after 15 days of storage [56]. Similarly, Souza et al. showed that a choline:citric acid (1:1) NaDES efficiently extracted phenolics from *Talisia esculenta*, and these extracts, when applied to fresh sausages, improved both oxidative stability and sensory attributes [57]. Collectively, these findings indicate that NaDES based on choline and organic acids can outperform conventional ethanol-based systems in terms of selectivity, efficiency, and potential sensory compatibility. Nevertheless, consumer perception studies and descriptive sensory analyses (such as triangle tests and preference mapping) [58] should be incorporated into future research to properly validate the acceptability of NaDES-containing food products in order to evaluate consumer acceptability and sensory descriptors, especially considering the possible contribution of lactic acid to sourness and of the choline-chloride-based system to overall taste and mouthfeel.

## 4. Conclusions

Based on the results of this study, NaDES proved to be more effective solvents than water or ethanol for extracting phenolic compounds. Among the formulations tested, the lactic-acid-based NaDES demonstrated the highest extraction yield and storage stability, with the glycerol-based formulation yielding similar results. Applying the extract to fresh-cut avocado, Lac alone contributed to maintaining phenolic stability, while LacEx provided additional enrichment during storage. Overall, lactic-acid-based NaDES represent a promising green solvent system for efficient phenolic extraction and for enhancing color stability in fresh-cut avocado, highlighting their potential for combined extraction and functional application. However, future studies should further assess the applicability of LacEx by comparing its anti-browning performance with commercial treatments and by evaluating its sensory impact on fresh-cut avocado.

## Figures and Tables

**Table 1:** Physicochemical characterization of the avocado peel flour. Values are expressed as mean ± standard deviation (SD).

Assessment	Parameter	Value (Mean ± SD)
Color	L*	40.81 ± 0.02
	a*	11.37 ± 0.03
	b*	27.24 ± 0.12
Water Activity	Aw	0.310 ± 0.006
pH	pH	5.48 ± 0.08
Particle Size	Dx 10 (µm)	222.8 ± 10.5
	Dx 50 (µm)	424.4 ± 18.1
	Dx 90 (µm)	736.6 ± 24.9

## Data Availability

The original contributions presented in this study are included in the article. Further inquiries can be directed to the corresponding author.

## Abbreviations

The following abbreviations are used in this manuscript:

Lac	Lactic-acid-based NaDES
LacEx	Lactic-acid-based NaDES extract
Gly	Glycerol-based NaDES
GlyEx	Glycerol-based NaDES extract
Cit	Citric-acid-based NaDES
CitEx	Citric-acid-based NaDES extract
Wa	Water
WaEx	Water extract
Et	Ethanol
EtEx	Ethanol extract
NaDES	Natural deep eutectic solvents
BA	Browning area
ΔE	Total color difference
HBA	Hydrogen bond acceptor
HBD	Hydrogen bond donor

## References

[1] Eurostat (2025). Food Waste and Food Waste Prevention—Estimates.

[2] Silva S.d.O., Mafra A.K.C., Pelissari F.M., Rodrigues de Lemos L., Molina G. (2025). Biotechnology in Agro-Industry: Valorization of Agricultural Wastes, By-Products and Sustainable Practices. Microorganisms.

[3] Giannakourou M.C., Tsironi T.N. (2021). Application of Processing and Packaging Hurdles for Fresh-Cut Fruits and Vegetables Preservation. Foods.

[4] Tarazona-Díaz M.P., Aguayo E. (2013). Assessment of By-Products from Fresh-Cut Products for Reuse as Bioactive Compounds. Food Sci. Technol. Int..

[5] Mármol I., Quero J., Ibarz R., Ferreira-Santos P., Teixeira J.A., Rocha C.M.R., Pérez-Fernández M., García-Juiz S., Osada J., Martín-Belloso O. (2021). Valorization of Agro-Food by-Products and Their Potential Therapeutic Applications. Food Bioprod. Process..

[6] Dey T., Bhattacharjee T., Nag P., Ritika, Ghati A., Kuila A. (2021). Valorization of Agro-Waste into Value Added Products for Sustainable Development. Bioresour. Technol. Rep..

[7] Mora-Sandí A., Ramírez-González A., Castillo-Henríquez L., Lopretti-Correa M., Vega-Baudrit J.R. (2021). Persea Americana Agro-Industrial Waste Biorefinery for Sustainable High-Value-Added Products. Polymers.

[8] Nascimento A.P.S., Duarte M.E.M., Rocha A.P.T., Barros A.N. (2025). Valorization of Avocado (*Persea americana*) Peel and Seed: Functional Potential for Food and Health Applications. Antioxidants.

[9] Grisales-Mejía J.F., Cedeño-Fierro V., Ortega J.P., Torres-Castañeda H.G., Andrade-Mahecha M.M., Martínez-Correa H.A., Álvarez-Rivera G., Mendiola J.A., Cifuentes A., Ibañez E. (2024). Advanced NADES-Based Extraction Processes for the Recovery of Phenolic Compounds from Hass Avocado Residues: A Sustainable Valorization Strategy. Sep. Purif. Technol..

[10] Alara O.R., Abdurahman N.H., Ukaegbu C.I. (2021). Extraction of Phenolic Compounds: A Review. Curr. Res. Food Sci..

[11] Martinović M., Krgović N., Nešić I., Žugić A., Tadić V.M. (2022). Conventional vs. Green Extraction Using Natural Deep Eutectic Solvents—Differences in the Composition of Soluble Unbound Phenolic Compounds and Antioxidant Activity. Antioxidants.

[12] Siamandoura P., Tzia C. (2023). Comparative Study of Novel Methods for Olive Leaf Phenolic Compound Extraction Using NADES as Solvents. Molecules.

[13] Gidado M.J., Gunny A.A.N., AlNashef I.M. (2025). Deep Eutectic Solvents as Green Alternatives in Postharvest Fruit Preservation: A Comprehensive Review. Food Bioprocess Technol..

[14] El Achkar T., Greige-Gerges H., Fourmentin S. (2021). Basics and Properties of Deep Eutectic Solvents: A Review. Environ. Chem. Lett..

[15] Morgana N.M., Magdalena E., Fernandez M.d.l.A., Fernanda S.M. (2022). NADES for Food Industry Innovation: Novel Bioadditives Based on Olive Oil Byproducts. Food Bioprod. Process..

[16] Sicaire A.-G., Filly A., Vian M., Fabiano-Tixier A.-S., Chemat F. (2018). Cosmo-RS-Assisted Solvent Screening for Green Extraction of Natural Products. Handbook of Green Chemistry.

[17] Molina-Hernandez J.B., Tappi S., Giannini G., Martinez-Correa H.A., Vanegas-Mahecha P. (2025). Comparison of Ultrasound-Assisted Extraction of Pumpkin Seed Oil (*Curcubita mochata*) with Conventional Extraction Methods: Response Surface Methodology for the Optimizing of Ultrasound-Assisted Extraction. Food Anal. Methods.

[18] Santos-Martín M., Cubero-Cardoso J., González-Domínguez R., Cortés-Triviño E., Sayago A., Urbano J., Fernández-Recamales Á. (2023). Ultrasound-Assisted Extraction of Phenolic Compounds from Blueberry Leaves Using Natural Deep Eutectic Solvents (NADES) for the Valorization of Agrifood Wastes. Biomass Bioenergy.

[19] de Albuquerque L.A., dos Reis M.L.C.M., Santana D.d.A., Oliva S.T., Silva M.F., Espino M., Gomez F.J.V., Dias F.d.S. (2025). Optimization of Natural Deep Eutectic Solvent Composition for Ultrasound-Assisted Extraction of Phenolic Compound from Propolis Samples. J. Chromatogr. B.

[20] Ozkan G. (2024). Valorization of Artichoke Outer Petals by Using Ultrasound-Assisted Extraction and Natural Deep Eutectic Solvents (NADES) for the Recovery of Phenolic Compounds. J. Sci. Food Agric..

[21] Della Posta S., Gallo V., Ascrizzi A.M., Gentili A., De Gara L., Dugo L., Fanali C. (2023). Development of a Green Ultrasound-Assisted Procedure for the Extraction of Phenolic Compounds from Avocado Peel with Deep Eutectic Solvents. Green Anal. Chem..

[22] Gómez-Urios C., Viñas-Ospino A., Puchades-Colera P., López-Malo D., Frígola A., Esteve M.J., Blesa J. (2022). Sustainable Development and Storage Stability of Orange By-Products Extract Using Natural Deep Eutectic Solvents. Foods.

[23] Grillo G., Gunjević V., Radošević K., Redovniković I.R., Cravotto G. (2020). Deep Eutectic Solvents and Nonconventional Technologies for Blueberry-Peel Extraction: Kinetics, Anthocyanin Stability, and Antiproliferative Activity. Antioxidants.

[24] Ivanović M., Krajnc P., Mlinarič A., Razboršek M.I. (2022). Natural Deep Eutectic Solvent-Based Matrix Solid Phase Dispersion (MSPD) Extraction for Determination of Bioactive Compounds from Sandy Everlasting (*Helichrysum arenarium* L.): A Case of Stability Study. Plants.

[25] Jovanović M.S., Krgović N., Živković J., Stević T., Zdunić G., Bigović D., Šavikin K. (2022). Ultrasound-Assisted Natural Deep Eutectic Solvents Extraction of Bilberry Anthocyanins: Optimization, Bioactivities, and Storage Stability. Plants.

[26] da Silva D.T., Smaniotto F.A., Costa I.F., Baranzelli J., Muller A., Somacal S., Monteiro C.S.A., Vizzotto M., Rodrigues E., Barcia M.T. (2021). Natural Deep Eutectic Solvent (NADES): A Strategy to Improve the Bioavailability of Blueberry Phenolic Compounds in a Ready-to-Use Extract. Food Chem..

[27] Manuela P., Drakula S., Cravotto G., Verpoorte R., Hruškar M., Radojčić Redovniković I., Radošević K. (2020). Biological Activity and Sensory Evaluation of Cocoa By-Products NADES Extracts Used in Food Fortification. Innov. Food Sci. Emerg. Technol..

[28] Braham F., Souza H.K.S., Magalhães J.M.C.S., Zaidi F., Gonçalves M.P. (2026). Enhancing the Quality and Shelf Life of Strawberries: Study of Pectin-Based Films and Application of Coatings with Natural Deep Eutectic Solvents and Moringa Oleifera Leaf Extract. Int. J. Biol. Macromol..

[29] Conte R., Sepe F., Margarucci S., Costanzo E., Petillo O., Peluso G., Marcolongo L., Calarco A. (2025). Functional Plant-Based Beverage Fortified with Hazelnut Cuticle Polyphenols: Antioxidant and Phenolic Content Characterization. Molecules.

[30] Domínguez-Rodríguez G., Amador-Luna V.M., Castro-Puyana M., Ibáñez E., Marina M.L. (2025). Sustainable Strategies to Obtain Bioactive Compounds from Citrus Peels by Supercritical Fluid Extraction, Ultrasound-Assisted Extraction, and Natural Deep Eutectic Solvents. Food Res. Int..

[31] Coutinho N.M., Silveira M.R., Pimentel T.C., Freitas M.Q., Moraes J., Fernandes L.M., Silva M.C., Raices R.S.L., Ranadheera C.S., Borges F.O. (2019). Chocolate Milk Drink Processed by Cold Plasma Technology: Physical Characteristics, Thermal Behavior and Microstructure. LWT.

[32] Goyeneche R., Roura S., Ponce A., Vega-Gálvez A., Quispe-Fuentes I., Uribe E., Di Scala K. (2015). Chemical Characterization and Antioxidant Capacity of Red Radish (*Raphanus sativus* L.) Leaves and Roots. J. Funct. Foods.

[33] Toro-Uribe S., Ibañez E., Decker E.A., Villamizar-Jaimes A.R., López-Giraldo L.J. (2020). Food-Safe Process for High Recovery of Flavonoids from Cocoa Beans: Antioxidant and HPLC-DAD-ESI-MS/MS Analysis. Antioxidants.

[34] Schouten M.A., Genovese J., Tappi S., Di Francesco A., Baraldi E., Cortese M., Caprioli G., Angeloni S., Vittori S., Rocculi P. (2020). Effect of Innovative Pre-Treatments on the Mitigation of Acrylamide Formation in Potato Chips. Innov. Food Sci. Emerg. Technol..

[35] Hossen M.S., Sotome I., Nanayama K., Sasaki T., Okadome H. (2016). Functional Properties of Submicron-Scale Rice Flour Produced by Wet Media Grinding. Cereal Chem..

[36] Popovic B.M., Micic N., Potkonjak A., Blagojevic B., Pavlovic K., Milanov D., Juric T. (2022). Novel Extraction of Polyphenols from Sour Cherry Pomace Using Natural Deep Eutectic Solvents—Ultrafast Microwave-Assisted NADES Preparation and Extraction. Food Chem..

[37] Bertolo M.R.V., Martins V.C.A., Plepis A.M.G., Bogusz S. (2021). Utilization of Pomegranate Peel Waste: Natural Deep Eutectic Solvents as a Green Strategy to Recover Valuable Phenolic Compounds. J. Clean. Prod..

[38] Rodríguez-Juan E., Rodríguez-Romero C., Fernández-Bolaños J., Florido M.C., Garcia-Borrego A. (2020). Phenolic Compounds from Virgin Olive Oil Obtained by Natural Deep Eutectic Solvent (NADES): Effect of the Extraction and Recovery Conditions. J. Food Sci. Technol..

[39] Fuad F., Nadzir M., Kamaruddin A. (2021). Hydrophilic Natural Deep Eutectic Solvent: A Review on Physicochemical Properties and Extractability of Bioactive Compounds. J. Mol. Liq..

[40] Esparza I., Cimminelli M.J., Moler J.A., Jiménez-Moreno N., Ancín-Azpilicueta C. (2020). Stability of Phenolic Compounds in Grape Stem Extracts. Antioxidants.

[41] Ferreyra S., Bottini R., Fontana A. (2023). Temperature and Light Conditions Affect Stability of Phenolic Compounds of Stored Grape Cane Extracts. Food Chem..

[42] Singleton V.L., Rossi J.A. (1965). Colorimetry of Total Phenolics with Phosphomolybdic-Phosphotungstic Acid Reagents. Am. J. Enol. Vitic..

[43] Velderrain-Rodríguez G.R., Quero J., Osada J., Martín-Belloso O., Rodríguez-Yoldi M.J. (2021). Phenolic-Rich Extracts from Avocado Fruit Residues as Functional Food Ingredients with Antioxidant and Antiproliferative Properties. Biomolecules.

[44] Kosińska A., Karamać M., Estrella I., Hernández T., Bartolomé B., Dykes G.A. (2012). Phenolic Compound Profiles and Antioxidant Capacity of Persea Americana Mill. Peels and Seeds of Two Varieties. J. Agric. Food Chem..

[45] Rodríguez-Martínez B., Ferreira-Santos P., Alfonso I.M., Martínez S., Genisheva Z., Gullón B. (2022). Deep Eutectic Solvents as a Green Tool for the Extraction of Bioactive Phenolic Compounds from Avocado Peels. Molecules.

[46] Whitaker J.R., Lee C.Y. (1995). Recent Advances in Chemistry of Enzymatic Browning.

[47] Măntăilă S., Aprodu I., Milea A.Ș., Balan N., Geana E.I., Râpeanu G., Stănciuc N. (2026). Advancements in Green Extraction of Polyphenols from Fetească Albă Grape Pomace Using Natural Deep Eutectic Solvents: Optimization and Applications as Inhibitors for Enzymatic Browning. Food Bioprocess Technol..

[48] Moon K.M., Kwon E.B., Lee B., Kim C.Y. (2020). Recent Trends in Controlling the Enzymatic Browning of Fruit and Vegetable Products. Molecules.

[49] Xu Y., Xiao S., Liu Y., Wang Y., Hou S., Teng J. (2025). The Stability of Polyphenol Oxidase in Deep Eutectic Solvents: Insights from Spectroscopy and Molecular Docking. Int. J. Biol. Macromol..

[50] Arias E., González J., Oria R., Lopez-Buesa P. (2007). Ascorbic Acid and 4-Hexylresorcinol Effects on Pear PPO and PPO Catalyzed Browning Reaction. J. Food Sci..

[51] Everette J.D., Bryant Q.M., Green A.M., Abbey Y.A., Wangila G.W., Walker R.B. (2010). Thorough Study of Reactivity of Various Compound Classes toward the Folin−Ciocalteu Reagent. J. Agric. Food Chem..

[52] Toivonen P.M.A., Brummell D.A. (2008). Biochemical Bases of Appearance and Texture Changes in Fresh-Cut Fruit and Vegetables. Postharvest Biol. Technol..

[53] Hu W., Sarengaowa W., Guan Y., Feng K. (2022). Biosynthesis of Phenolic Compounds and Antioxidant Activity in Fresh-Cut Fruits and Vegetables. Front. Microbiol..

[54] Pace B., Cefola M., Renna F., Attolico G. (2011). Relationship between Visual Appearance and Browning as Evaluated by Image Analysis and Chemical Traits in Fresh-Cut Nectarines. Postharvest Biol. Technol..

[55] Ameen S.M., Caruso G. (2017). Lactic Acid in the Food Industry.

[56] Ran H., Li H., Peng D., Hou Y., Jiang Y., Kuang J., Wang A., Zhang X., Wang G. (2025). Research Progress on Extraction of Flavonoids with Deep Eutectic Solvents from Natural Products. J. Mol. Liq..

[57] Souza P.d.S., Cardoso F.A.R., Lima M.V.d.S., Cristaldo Heck S., Marques L.L.M. (2024). Extraction of Bioactive Compounds from Peel and Seeds of Pitomba (*Talisia esculenta*) Using Eutectic Solvents. Int. J. Food Sci. Technol..

[58] Martins K.P., Hernandez Brito N.L., Costa G.B., Silva Ramos J., Silvestre Barbosa Alessi A.C., Santos T.A., Melo da Silva A.E., Machado I.F., Medeiros Marques L.L., Droval Arcain A.A. (2025). Natural Deep Eutectic Solvents (NADES) in Food Systems: Emerging Applications, Extraction Efficiency, Safety Concerns, and Regulatory Challenges. J. Agric. Food Chem..

